# Anxiety and Depression in Health Workers and General Population During COVID‐19 in IRAN: A Cross‐Sectional Study

**DOI:** 10.1002/npr2.12153

**Published:** 2020-12-25

**Authors:** Leila Hassannia, Fatemeh Taghizadeh, Mahmood Moosazadeh, Mehran Zarghami, Hassan Taghizadeh, Azadeh Fathi Dooki, Mohammad Fathi, Reza Alizadeh‐Navaei, Akbar Hedayatizadeh‐Omran, Niloufar Dehghan

**Affiliations:** ^1^ Faculty of Health Mazandaran University of Medical Sciences Sari Iran; ^2^ Student Research Committee, Psychiatry and Behavioral Sciences Research Center Addiction Institute Mazandaran University of Medical Sciences Sari Iran; ^3^ Gastrointestinal Cancer Research Center Non‐communicable Diseases Institute Mazandaran University of Medical Sciences Sari Iran; ^4^ Department of Psychiatry School of Medicine & Psychiatry and Behavioral Sciences Research Center Addiction Institute Mazandaran University of Medical Sciences Sari Iran; ^5^ Anesthesiology Research Center Shahid Beheshti University of Medical Sciences Tehran Iran; ^6^ Departman of psychology Islamic Azad University Sari branch; ^7^ Gastrointestinal Cancer Research Center Mazandaran University of Medical Sciences Sari Iran; ^8^ Niloufar Dehghan Tehran University Tehran Iran

**Keywords:** anxiety, COVID‐19, depression, doctor, nurse

## Abstract

**Background:**

The COVID‐19 outbreak has exerted a great deal of psychological pressure on Iranian health workers and the general population. The aim of this study was to determine the effect of pandemic on anxiety and depression in Iranian population.

**Methods:**

An online cross‐sectional study was conducted for the general public and healthcare workers in Iran using a questionnaire comprised of demographic questions and Hospital Anxiety and Depression Scale. Chi‐square test and univariate and multivariate logistic regression models were conducted.

**Results:**

Of the 2045 participants, 1136 (65.6%) were considered to have moderate and severe anxiety symptoms, and 865 (42.3%) had moderate and severe depression symptoms. The prevalence of anxiety was higher in the females than in the males (OR = 1.4, 95% CI: 1.123‐1.643, *P *= .002); the prevalence of anxiety was significantly higher in those aged 30‐39 years than in other age‐groups (OR = 1.6, 95% CI: 1.123‐2.320, *P* = .001); furthermore, the prevalence of anxiety and depression was significantly higher in doctors and nurses compared with other occupations ((OR = 1.9, 95% CI: 1.367‐2.491, *P* < .001) and (OR = 1.5, 95% CI: 1.154‐2.021, *P* = .003)). In addition, the prevalence of anxiety symptoms in the likely infected COVID‐19 group was higher than in the noninfected COVID‐19 group (OR = 1.35, 95% CI: 1.093‐1.654, *P* = .005).

**Conclusions:**

Regarding the high prevalence of anxiety and depression symptoms, especially among healthcare workers, appropriate psychological/psychiatric intervention necessitates.

## Abstract 2

An online cross‐sectional study was conducted for the general public and healthcare workers in Iran using a questionnaire comprised of demographic questions and Hospital Anxiety and Depression Scale. Regarding the high prevalence of anxiety and depression symptoms, especially among healthcare workers, appropriate psychological/psychiatric intervention necessitates.

## Graphical abstract

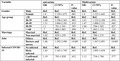

## 1 INTRODUCTION

COVID‐19 is an acute respiratory illness with unknown causes that began in December 2019 in Wuhan, Hubei Province, China.^1^ In addition to causing physical injuries, this disease has seriously impacted the human mental health.^2^ On January 20, China confirmed the transfer of COVID‐19 from human to human.^3^ Since then, people have shown behaviors associated with anxiety. Those in quarantined areas may experience boredom, anger, and loneliness. The symptoms of a viral infection such as cough and fever may cause cognitive distress and anxiety.^4^, ^5^, ^6^ Several mental disorders such as depression, panic attack, delirium, suicidal thoughts, anxiety, and psychotic symptoms were reported during the early stages of SARS disease.^4^ During the COVID‐19 epidemic and SARS disease in China, major psychological health issues were observed in young people, those spending a great amount of time on the disease, and healthcare workers.^7^ Due to the increase in the virus‐related mortalities, both the medical staff and the general public^8^ have experienced psychological problems such as anxiety and depression.^9^ On average, the medical personnel work for 16 to 22 hours per day, putting their mental health at risk.^10^ High‐risk positions with constant contact with the infected and high‐risk‐infected environments with inadequate protection from contamination, lack of contact with family members, frustration, prejudice, loneliness, and fatigue further cause mental health issues such as stress, anxiety, depressive symptoms, insomnia, denial, anger, and fear.^9^, ^11^ Mental health problems not only affect the medical staff's attention, understanding, and decision‐making ability, but may also prevent them from combatting the virus.^9^ Many of the medical and nursing staff working in Wuhan had mental health disorders.^12^ Medical staff not only tolerate the excess workload, but also run a high risk of infection. Furthermore, prolonged stress increases susceptibility to depression or other mental illnesses, leading to the increased risk of infection.^13^ Therefore, it is necessary to pay attention to protective factors and the process of successful adaptation to adverse conditions.^14^ The paucity of research in Iran, which is currently affected by this virus, has led researchers to investigate the effects of the pandemic on depression and anxiety in the general public and healthcare workers. Finally, it is important to establish early purposive mental health interventions throughout Iran.

## 2 METHODS

### 2.1 Settings and participants

The target population was the general Iranian population and health workers. Inclusion criteria were Iranian nationality, 15 years of age or more, willingness to participate, online Internet access, and ability to understand Persian language. Exclusion criteria were less than 15 years of age, major psychological disorders, and unwillingness to be studied. The project was conducted during quarantine in Iran, the time that transfers between provinces was limited, and almost socioeconomic activities were restricted from April 6 to April 15, 2020.

### 2.2 Informed consent

Informed consent was obtained electronically prior to data collection. To ensure the quality of the survey, a response range was considered for items such as age which was restricted to 15‐80 years, and the respondents were encouraged to carefully read the questionnaire instructions. Ultimately, a total of 2045 participants who completed the questionnaires were included in the data analysis.

### 2.3 Investigation approach

The study was conducted during quarantine, including interprovincial travel ban, prohibition of nonessential business activities, and closure of schools and universities. This was the first quarantine in Iran, and the reason for research in this period was to investigate the effect of the outbreak of COVID‐19 and the quarantine to prevent of the spread of this pandemic on people's mental health.

Electronic "Porseline" Questionnaire was used as the survey tool and a professional online survey platform which can be used for the questionnaire survey, evaluation, and other purposes. Participants were recruited via advertisements on social media such as WhatsApp, Telegram, LinkedIn, and Instagram.

### 2.4 Investigation tools

The present study made use of online demographic questionnaires and Hospital Anxiety and Depression Scale (HADS) for the general public and healthcare workers.^10^

#### 2.4.1 Demographic information

A self‐designed questionnaire was devised, comprising items related to the province of residence, age, gender, job, level of education, marital status, and an additional question about the infected individuals.

#### 2.4.2 Hospital anxiety and depression scale

This questionnaire was developed by the questionnaire consists of two subscales, one related to depression and the other associated with anxiety,^15^, ^16^ and it includes a total of 14 questions. This scale is used to classify participants into three categories: without anxiety and/or depression (scores of 0 to 7), possible anxiety and/or depression (scores ranging from 8 to 10), and anxiety and/or depression (scores varying from 11 to 21).^17^ This questionnaire was used on a general population of 6659 individuals aged 65‐80 in Sweden; the internal consistency was 0.92 for the anxiety subscale and 0.88 regarding the depression subscale; moreover, the Cronbach's alpha was 0.87 on the anxiety subscale and 0.81 on the depression scale.^18^ In Iran, the scale had an acceptable validity and reliability (Cronbach's alpha of 0.78 and 0.86 on anxiety (HADS‐S) and depression (HADS‐D), respectively).^19^, ^20^

### 2.5 Statistical analysis

Descriptive analyses were performed to describe the demographic characteristics of the Iranian population. The prevalence of anxiety and depression symptoms, stratified by gender, age, education, occupation, and infection with COVID‐19, was determined. Chi‐square test (*χ* ^2^) was used to compare the categorical variables, while independent sample t test and ANOVA test compared the continuous variables with normal distribution. Univariate and multivariate logistic regression models were conducted to explore the potential contributing factors for anxiety and depression symptoms during COVID‐19 outbreak. Odds ratio (*OR*) and 95% confidence interval were analyzed from logistic regression models. All data were analyzed using Statistical Package for Social Sciences (SPSS) version 24.0. *P*‐values of less than 0.05 were considered as statistically significant (two‐sided tests).

## 3 RESULTS

93% of the respondents completed the questionnaire on WhatsApp, 6% on Telegram, and 1% on other applications. The response rate was 59%, and the average time of response to questionnaire was 08.17 minutes. The results of this study are as follows:

### 3.1 General demographic data

Based on the online survey, 2045 qualified questionnaires were finally obtained. The characteristics of the participants are shown in Table 1. Following the analysis of 2045 questionnaires, the average age of the respondents was 44.07 ± 11.638 (years), ranging from 20 to 60 years old, of whom 181 (8.9%) were under 30, 536 (26.2%) were 30‐39 years old, 648 (31.6%) were 40‐49 years old, 505 (24.7%) were 50‐59 years old, and 176 (8.6%) were equal or above 60 years old. Of the 2045 participants, 127 )6.2(% were doctors, 105 )5.1(% were nurses, 229 (11.2%) were health staff, 26 (1.3%) were laboratory staff, and 580 (28.4%) were other employee staff. Moreover, 671 (32.8%) of the participants were males, 1374 (67.2%) were females, 1584 (77.5%) were married, 68 (3.3%) hold a PhD, 494 (24.2%) had a master of sciences, 738 (36.1%) were bachelors, 110 (5.4%) had associate degrees, 330 (16.1%) had a high school diploma, and 132 (6.5%) had lower education levels.

**Table 1:** Demographic characteristics of study sample (N = 2045)

Variables	n (%)
Gender	
Male	671 (32.5)
Female	1374 (67.2)
Education	
Under diploma	132 6.5)
Diploma	330 (16.1)
Associate degree	110 (5.4)
Bachelor	738 (36.1)
Master of science	494 (24.2)
Physician	126 (6.2)
Specialist physician	47 (2.3)
Ph.D	68 (3.3)
Marriage	
Married	1584 (77.5)
Unmarried	461 (22.5)
Age, Y	
<30	181 (8.9)
30‐39	536 (26.2)
40‐49	648 (31.6)
50‐59	505 (24.7)
>=60	176 (8.6)
Occupation	
Physician (doctor)	127 (6.2)
Nurse	105 (5.1)
Laboratory staff	26 (1.3)
Health staff	229 (11.2)
Employee of other jobs	580 (28.4)
Self‐employment	304 (14.9)
Housewife	434 (21.2)
Jobless	74 (3.6)
University or high school student	166 (8.1)

### 3.2 Prevalence of anxiety and depression symptoms

Tables 2 and 3 show the prevalence of anxiety and depression symptoms stratified by gender, education, marriage, age, and occupation. Univariate and multivariate logistic regression models are presented in Tables 5 and 6. Of the 2045 participants, 1136 (65.6%) had moderate and severe anxiety symptoms, and 865 (42.3%) had moderate and severe depression symptoms. According to Tables 5 and 6 and the logistic regression models, the prevalence of anxiety in females was significantly higher than in males (*OR* = 1.4, 95% *CI*: 1.123‐1.643, *P*=.002). However, there were no significant differences between males and females concerning the prevalence of depression (*OR* = 1.1, 95% *CI*: 0.912‐1.336, *P*=.312). The prevalence of anxiety in the age‐group 30‐39 was significantly higher than in other age‐groups (OR = 1.6, 95% CI: 1.123‐2.320, *P* = .01). The prevalence of anxiety and depression was significantly higher in doctors and nurses compared with other occupations ((OR = 1.9, 95% CI: 1.367‐2.491, *P* < .001) and (OR = 1.5, 95% CI: 1.154‐2.021, *P* = .003)). Other findings showed that the prevalence of anxiety and depression was not related to the marital status (*P*=.982, 0.354). Furthermore, the prevalence of anxiety in specialist physicians was significantly higher than in general physicians, and the anxiety of both groups was significantly higher than other education levels (X2 = 30.346, *P* = .007); however, the prevalence of depression was not significant in education levels (X2 = 22.800, *P* = .064).

**Table 2:** Prevalence of anxiety symptoms in participants during COVID‐19 outbreak in Iran population stratified by gender, education, marriage, age, and occupation (N = 2045)

Variables n (%)	Nonanxiety	Moderate anxiety	Anxiety	X 2	*P*
Gender					
Male	335) 49.9(	179)26.7(	157)23.4(	16.864	.001
Female	574 )41.8(	368 (26.8)	432 (31.4)		
Education					
Under diploma	75 (56.8)	28 (21.2)	29 (22.0)	30.346	.007
Diploma	150 (45.5)	87 (26.4)	93 (28.2)		
Associate degree	44 (40.0)	28 (25.5)	38 (24.5)		
Bachelor	304 (41.2)	206 (27.9)	228 (30.9)		
Master of science	236 (47.8)	124 (25.1)	134 (27.1)		
General physician	46 (36.5)	38 (30.2)	42 (33.3)		
Specialist physician	15 (31.9)	16 (34)	16 (34.0)		
Ph.D	39 (57.4)	20 (26.4)	9 (13.2)		
Marriage					
Married	932 (58.8)	329 (20.8)	323 (20.4)	3.723	.155
Unmarried	248 (53.8)	107 (23.2)	106 (23)		
Age, Y					
<30	92 (50.8)	43 (23.8)	46 (25.4)	45.486	.001
30‐39	204 (38.1)	142 (26.5)	190 (35.4)		
40‐49	263 (40.6)	181 (28)	203 (31.4)		
50‐59	245 (48.5)	138 (27.3)	122 (24.2)		
>=60	105 (59.7)	43 (24.4)	28 (15.9)		
Occupation					
Physician (doctor)	40 (31.5)	42 (33.1)	45 (35.4)	34.846	.004
Nurse	33 (31.4)	29 (27.6)	43 (41)		
Laboratory staff	11 (42.3)	7 (26.9)	8 (30.8)		
Health staff	97 (42.4)	61 (26.6)	71 (31.0)		
Employee of other jobs	290 (50.0)	143 (24.7)	147 (25.3)		
Self‐employment	132 (43.10)	78 (25.7)	94 (30.9)		
Housewife	187 (43.1)	126 (29.0)	121 (27.9)		
Jobless	30 (40.5)	20 (27.0)	24 (32.4)		
University or high school student	89 (53.6)	41(24.7)	36 (21.7)		

**Table 3:** Prevalence of depression symptoms in participants during COVID‐19 outbreak in Iran population stratified by gender, education, marriage, age, and occupation (N = 2045)

Variables n(%)	Nondepression (n = 62)	Moderate depression (n = 62)	Depression (n = 24)	X 2	*P*
Gender					
Male	403 (60.1)	138 (20.6)	130 (19.4)	2.453	0.293
Female	777 (56.6)	298 (21.7)	299 (21.8)		
Education					
Under diploma	79 (59.8)	31 (23.5)	22 (16.7)	22.800	0.064
Diploma	192 (58.2)	66 (20.0)	72 (21.8)		
Associate degree	56 (50.9)	24 (21.8)	30 (27.3)		
Bachelor	418 (56.6)	156 (21.1)	164 (22.2)		
Master of science	307 (62.1)	97 (19.6)	90 (18.2)		
General physician	60 (47.6)	37 (29.4)	29 (23.0)		
Specialist physician	22 (46.8)	11 (23.4)	14 (29.8)		
Ph.D	46(67.6)	14 (20.6)	8 (11.8)		
Marriage					
Married	932 (58.8)	329 (20.8)	323 (20.4)	3.723	0.155
Unmarried	248 (53.8)	107 (23.2)	106 (23.0)		
Age, Y					
<30	101 (55.8)	40 (22.1)	40 (22.1)	26.098	0.001
30‐39	280 (52.2)	129 (24.1)	127 (23.7)		
40‐49	369 (57.0)	127 (19.6)	151 (23.3)		
50‐59	309 (61.2)	115 (22.8)	81 (16.0)		
>=60	121 (68.8)	25 (14.2)	30 (17.0)		
Occupation					
Physician (Doctor)	61 (48.0)	32 (25.2)	34 (26.8)	28.165	0.30
Nurse	51 (48.6)	27 (25.7)	27 (25.7)		
Laboratory staff	15 (57.7)	6 (23.1)	5 (19.2)		
Health staff	125 (54.6)	49 (21.4)	55 (24.0)		
Employee of other jobs	367 (63.3)	110 (19.0)	103 (17.8)		
Self‐employment	176 (57.9)	61 (20.1)	67 (22.0)		
Housewife	252 (58.1)	96 (22.1)	86 (19.8)		
Jobless	43 (58.1)	9 (12.2)	22 (29.7)		
University or high school student	90 (54.2)	46 (27.7)	30 (18.1)		

### 3.3 Prevalence of anxiety and depression symptoms in the COVID‐19 infected

The results of the analysis of the infected are shown in Table 4, 5, and 6. The prevalence of anxiety symptoms in likely infected COVID‐19 group was higher than in the noninfected COVID‐19 group (OR = 1.35, 95% CI: 1.093‐1.654, *P* = .005), and compared to the infected group, it was not statistically significant (OR = 1.13, 95% CI: 0.734‐1.744, *P*=.577). Meanwhile, the prevalence of depression symptoms in the likely infected COVID‐19 group was not statistically significant compared with the noninfected (OR = 1.2, 95% CI: 0.988‐1.483, *P*=.066) and infected (OR = 1.46, 95% CI: 0.956‐2.239, *P*=.080) groups.

**Table 4:** Prevalence of infected people and the relationship to anxiety and depression symptoms

Virus infection status Variables n (%)	I was infected with the virus and was treated (confirmed infected)	I was infected with the virus and being under the treatment (confirmed infected)	I was likely infected with the virus and recovered with the symptoms of a cold (likely infected)	I have never been infected with this virus (no infected)	X^2^	*P*
Anxiety						
Nonanxiety	28 (40.6)	12 (48.0)	206 (38.3)	663 (46.9)	23.373	.001
Moderate anxiety	17 (24.6)	5 (20.0)	138 (25.7)	387 (27.4)		
Anxiety	24 (34.8)	8 (32.0)	194 (36.1)	363 (25.7)		
Depression						
Nondepression	36 (52.2)	11 (44.0)	291 (54.1)	842 (59.6)	11.457	.075
Moderate depression	12 (17.4)	6 (24.0)	129 (24.0)	289 (20.5)		
Depression	21 (30.4)	8 (32.0)	118 (21.9)	282 (20.0)		
Total 2045(100)	69 (3.4)	25 (1.2)	538 (26.3)	1413 (69.1)		

**Table 5:** Results of univariate and multivariate logistic regression analyses of anxiety symptoms in participants (N = 2045)

Variable		Univariate			Multivariate		
		OR	CI 95%	*P*‐value	OR	CI 95%	*P*‐value
Gender	Male	Ref.	Ref.	Ref.	Ref.	Ref.	Ref.
	Female	1.4	1.154‐1.673	.001	1.4	1.123‐1.643	.002
Age‐group	<30	Ref.	Ref.	Ref.	Ref.	Ref.	Ref.
	30‐39	1.68	1.198‐2.363	.003	1.61	1.123‐2.320	.010
	40‐49	1.51	1.084‐2.101	.015	1.47	1.008‐2.131	.045
	50‐59	1.1	0.781‐1.541	.593	1.04	0.704‐1.542	.837
	>=60	0.7	0.460‐1.063	.094	0.7	0.443‐1.114	.133
Marriage	Married	Ref.	Ref.	Ref.	Ref.	Ref.	Ref.
	Nonmarried	1.02	0.829‐1.259	.838	0.1	0.780‐1.275	.982
Jobs	Others	Ref.	Ref.	Ref.	Ref.	Ref.	Ref.
	Doctors and nurses	1.86	1.392‐2.496	<.001	1.85	1.367‐2.491	<.001
Infected COVID‐19	No infected	Ref.	Ref.	Ref.	Ref.	Ref.	Ref.
	Likely infected	1.43	1.163‐1.745	.001	1.35	1.093‐1.654	.005
	Confirmed infected	1.19	0.783‐1.820	.412	1.13	0.734‐1.744	.577

**Table 6:** Results of univariate and multivariate logistic regression analyses of depression symptoms in participants (N = 2045)

Variable		Univariate			Multivariate		
		OR	CI 95%	*P*‐value	OR	CI 95%	*P*‐value
Gender	Male	Ref.	Ref.	Ref.	Ref.	Ref.	Ref.
	Female	1.155	0.958‐1.394	.132	1.1	0.912‐1.336	.312
Age‐group	<30	Ref.	Ref.	Ref.	Ref.	Ref.	Ref.
	30‐39	1.154	0.823 ‐1.620	.406	1.17	0.815‐1.679	.394
	40‐49	0.951	0.682‐1.326	.951	0.98	0.672‐1.417	.898
	50‐59	0.801	0.568‐1.129	.801	0.81	0.546‐1.199	.291
	>=60	0.574	0.372‐0.885	.574	0.61	0.378‐0.968	.036
Married	Marriage	Ref.	Ref.	Ref.	Ref.	Ref.	Ref.
	Nonmarriage	1.228	0.996‐1.513	.054	1.1	0.881‐1.427	.354
Jobs	Others	Ref.	Ref.	Ref.	Ref.	Ref.	Ref.
	Doctors and nurses	1.536	1.168‐2.020	.002	1.53	1.154‐2.021	.003
Infected COVID‐19	No infected	Ref.	Ref.	Ref.	Ref.	Ref.	Ref.
	Likely infected	1.252	1.025‐1.529	.028	1.2	0.988‐1.483	.066
	Confirmed infected	1.475	0.971‐2.240	.069	1.46	0.956‐2.239	.080

Abbreviations: 95% *CI*, 95% confidence interval; and Ref., reference group; COVID‐19, 2019 coronavirus disease; *OR*, odds ratio.

## 4 DISCUSSION

The aim of this study was to investigate the anxiety and depression symptoms in health workers and the general public during COVID‐19 pandemic in Iran.

Based on the results of the current study, 65.6% of the participants had anxiety symptoms, and 42.3% had depression symptoms. Furthermore, women had more anxiety than men, and anxiety in those aged 30‐39 was significantly higher than in other age‐groups. Anxiety and depression were significantly more prevalent in doctors and nurses compared with other occupations. Additionally, anxiety symptoms in the likely infected COVID‐19 group were higher than in the noninfected.

Previous studies reported emotional distress severity and symptoms of stress and anxiety in many people and health workers. The following are some of these studies:

### 4.1 Anxiety and depression symptoms in general population

Lee SM et al made use of interviews and HADS and found that 11% of the participants were anxious, and 15.1% were depressed in the early stages of the outbreak.^21^ Cao et al examined the psychological impacts of the COVID‐19 pandemic on the students of a Chinese university; they reported that 0.9% of the students had severe anxiety, 21.3% had mild anxiety, and 2.7% had moderate anxiety.^22^ In the present study, 24.7% of the students had moderate anxiety, and 21.7% had depression symptoms. Guo et al^23^ reported a high prevalence of mental health problems in the general population during COVID‐19. In a study on residual psychiatric disorders among the survivors of SARS, 25% of the patients were found to have PTSD symptoms, while 15.6% had severe depression.^5^ Furthermore, Jeong H et al found that 16.6% and 7.6% of the uninfected individuals in quarantine had feelings of anger and anxiety, respectively,^24^ whereas 53.1% of the uninfected participants had anxiety symptoms in the present study. Wang C et al in a study on 1,210 respondents from 194 Chinese cities, 53.8% of the respondents rated the psychological impacts of the outbreak as moderate or severe. 16.5% reported moderate to severe depressive symptoms, and 28.8% reported moderate to severe anxiety symptoms. Females, students, and those with physical symptoms and poor health had significantly higher levels of anxiety and depression.^25^ This is in line with a systematic review where students, females, and individuals with the symptoms of COVID‐19 had higher rates of depression and anxiety.^26^ 65.6% of the participants in our study had anxiety symptoms, and 42.3% had depression symptoms. The odds ratio of anxiety symptoms in females was 1.4 times more than that of male. Huang Y and Zhao N investigated 7,236 volunteers and reported that the overall prevalence of depressive symptoms in the general public was 20.1%. Moreover, young people had a significantly higher prevalence of depression symptoms in comparison with others.^27^ The multivariate logistic regression in our results revealed that the prevalence of depression symptoms in the age‐group >=60 years and the anxiety symptoms in those aged 30‐39 years were higher than others.

### 4.2 Anxiety and depression symptoms in healthcare workers

The physicians showed higher scores of anxiety and depression compared with other medical staff.^4^ Mo Y et al found that stress of 180 nurses taking care of COVID‐19 patients was 39.91 ± 12.92 with a score of 39.91%.^28^ Guo et al studied the psychological impact of COVID‐19 pandemic on 11 118 Chinese hospital staff. They observed that despite the insignificant psychological impact of COVID‐19 on the medical staff in China, it was necessary to take measures to protect them from the adverse effects of COVID‐19.^29^ The results of SARS outbreak showed that 18%‐57% of the treatment staff experienced emotional distress in the entire period of infection.^30^ Huang et al studied the outbreak of generalized anxiety disorder and the depressive symptoms of COVID‐19 on 7,236 Chinese people; their results revealed the significant impact of COVID‐19 pandemic on the mental health of the participants.^31^

Zhang et al investigated the effects of psychiatric crisis intervention on the medical staff and patients' families in COVID‐19 hospitals. They reported that the intervention was conducive to reducing social anxiety during the pandemic.^1^ Previous studies reported that the mental health of medical workers was in a worse state compared with the general population,^32^, ^33^, ^34^ which is consistent with the present results.

Zhu J et al found that the prevalence of anxiety and depression symptoms was 11.4% and 45.6% in physicians and 27.9% and 43.0% in nurses.^35^ In a study in Singapore and India, the depression symptoms were reported 10.6% and anxiety symptoms were reported 15.7% in medical workers.^36^

In the current study, 51.4% and 52% of the nurses and doctors had depression symptoms, while 68.6% and 68.5% had anxiety symptoms. The nurses and doctors in Iran have experienced higher symptoms of anxiety and depression than in the China^35^ and other countries^36^ during the outbreak. Perhaps, one of the reasons is the lack of sufficient personal protective equipment such as gloves and masks at the beginning of the outbreak.

Among the 994 medical and nursing staff in Wuhan, 36.9% had subthreshold mental health disorders, 34.4% had mild disorders, 22.4% had moderate disorders, and 6.2% had severe disorders.^12^ Huang Y and Zhao N found that compared with other occupational groups, healthcare workers were likely to have a lower quality of sleep.^27^

Zandifar and Badrfam ^37^ stated that the severity, uncertainty, and unpredictability of COVID‐19, social quarantine, and misinformation contributed to anxiety. They further highlighted the need for both mental health services, particularly for vulnerable populations, and the establishment of social wealth to reduce the adverse psychological effects of the outbreak.

### 4.3 Anxiety and depression symptoms in infected COVID‐19 people

In our study, Anxiety symptoms in suspected COVID‐19 people were higher than the no infected COVID‐19 group and confirmed infected group. While, depression symptoms in suspected COVID‐19 people were not statically significant comparing to the no infected and infected groups. Nguyen HC et al. found that the people with Suspected COVID‐19 symptoms had a higher depression likelihood than those without COVID‐19.^38^

## 5 CONCLUSIONS AND RECOMMENDATIONS

The majority of the medical staff and general population in our study experienced anxiety and depression symptoms. To address the concerns about COVID‐19 in Iran, a free 1480 hotline has been provided, mental health professionals are providing guidelines in hospitals and medical centers, and NGO groups consisting of psychologists are counseling for free. Healthcare providers can also offer suggestions for coping with anxiety and depression and link patients to social and mental health services. Since media reports can be emotionally distressing, COVID‐related news should be checked and restricted. Moreover, to improve the mental health of health workers, training in coping techniques such as relaxation and stress management skills is recommended. Physical health education training and telephone counseling by specialist psychologists are also required. Researchers should also assess the effects of COVID‐19 on other vulnerable people, including children and adolescents, those residing in rural areas and lacking access to health care, and individuals belonging to lower socioeconomic states. It is also necessary to develop mental health interventions which are time‐limited, sensitive to culture, and can be taught to volunteers and healthcare workers.

## 6 LIMITATIONS

In this cross‐sectional study, we were not able to establish a causal link; furthermore, we used the self‐rating scale to evaluate the depression and anxiety symptoms of the medical staff and the general public. Finally, the age‐group did not include those above 60 years of age possibly due to the lack of access to Internet smartphones, or applications.

## CONFLICT OF INTEREST

None to declare.

## AUTHOR CONTRIBUTIONS

FT conceptualized and designed the study, review and revised the manuscript, and approved the final manuscript as submitted. LH, RA, and AH designed the data collection instruments, coordinated and supervised data collection, carried out the initial analyses, and interpreted the data, drafted the initial manuscript, and approved the final manuscript as submitted. All of the authors agree to be cooperative for all aspects of the study and approved the manuscript.

## FUNDING INFORMATION

This study was supported by Mazandaran University of Medical Sciences.

## APPROVAL OF THE RESEARCH PROTOCOL BY AN INSTITUTIONAL REVIEWER BOARD

The protocol for this research project has been approved by a suitably constituted Ethics Committee of the institution and it conforms to the provisions of the Declaration of Helsinki. Committee of Mazandaran University of Medical sciences, Approval No. IR.MAZUMS.REC.1399.283.

## INFORMED CONSENT

Electronic informed consent was obtained from all participants.

## ETHICAL CONSIDERATION

All procedures were conducted according to the Declaration of Helsinki and were approved by the Ethics Committee of Mazandaran University of Medical Sciences. The participants could leave without any explanation before the research.

## Data Availability

The raw data belonged to the present study cannot be made publicly available, because the disclosure of personal data was not included in the research protocol of the present study.

## References

[1] Wang D , Hu B , Hu C , Zhu F , Liu X , Zhang J , et al. Clinical characteristics of 138 hospitalized patients with 2019 novel coronavirus–infected pneumonia in Wuhan, China. JAMA. 2020;323:1061–9. 32031570 10.1001/jama.2020.1585 PMC7042881

[2] Gao W , Ping S , Liu X . Gender differences in depression, anxiety, and stress among college students: a longitudinal study from China. J Affective Disord. 2020;263:292–300. 10.1016/j.jad.2019.11.121 31818792

[3] Zhang G , Zhang J , Wang B , Zhu X , Wang Q , Qiu S . Analysis of clinical characteristics and laboratory findings of 95 cases of 2019 novel coronavirus pneumonia in Wuhan, China: a retrospective analysis. Respiratory Research. 2020;21:1. 10.1186/s12931-020-01338-8 PMC7099829 32216803

[4] Xiang Y‐T , Yang Y , Li W , Zhang L , Zhang Q , Cheung T , et al. Timely mental health care for the 2019 novel coronavirus outbreak is urgently needed. Lancet Psychiatry. 2020;7:228–9. 32032543 10.1016/S2215-0366(20)30046-8 PMC7128153

[5] Chen Q , Liang M , Li Y , Guo J , Fei D , Wang L , et al. Mental health care for medical staff in China during the COVID‐19 outbreak. Lancet Psychiatry. 2020;7:e15–e16. 32085839 10.1016/S2215-0366(20)30078-X PMC7129426

[6] Taghizadeh F , Taghizadeh H . Naso‐pharyngeal discharge: The first symptom of COVID‐19 infection: Report two cases from Iran. Clinical Case Reports. 2020;8. 10.1002/ccr3.3214 PMC7436221 32837726

[7] Mak IWC , Chu CM , Pan PC , Yiu MGC , Chan VL . Long‐term psychiatric morbidities among SARS survivors. General Hosp Psychiatry. 2009;31:318–26. 10.1016/j.genhosppsych.2009.03.001 PMC7112501 19555791

[8] Taghizadeh F , Cherati JY . Procrastination and Self-Efficacy Among Intravenous Drug Users on a Methadone Maintenance Program in Sari City, Iran, 2013. Iranian journal of psychiatry and behavioral sciences. 2015;9. 10.17795/ijpbs-3738 PMC4733314 26834810

[9] Kang L , Li Y , Hu S , Chen M , Yang C , Yang BX , et al. The mental health of medical workers in Wuhan, China dealing with the 2019 novel coronavirus. Lancet Psychiatry. 2020;7:e14. 32035030 10.1016/S2215-0366(20)30047-X PMC7129673

[10] Majidi H , Bani‐Mostafavi E‐S , Mardanshahi Z , Godazandeh F , Ghasemian R , Heydari K , Alizadeh‐Navaei R . High‐resolution computed tomography finding in 552 patients with symptomatic COVID‐19: first report from north of Iran. Emergency Radiology. 2020;27(6):633–639. 10.1007/s10140-020-01819-9 32661945 PMC7355135

[11] Zarghami M , Taghizadeh F , Sharifpour A , Alipour A . Efficacy of guided self‐change for smoking cessation in chronic obstructive pulmonary disease patients: A randomized controlled clinical trial. Tobacco Induced Dis. 2019;17:90. 10.18332/tid/114227 PMC6915435 31892920

[12] Kang L , Ma S , Chen M , Yang J , Wang Y , Li R , et al. Impact on mental health and perceptions of psychological care among medical and nursing staff in Wuhan during the 2019 novel coronavirus disease outbreak: A cross‐sectional study. Brain Behav Immun. 2020;S0889–1591(0820):30348–30342. 10.1016/j.bbi.2020.03.028 PMC7118532 32240764

[13] Liao Y‐T , Hsieh M‐H , Yang Y‐H , Wang Y‐C , Tsai C‐S , Chen VC‐H , et al. Association between depression and enterovirus infection: A nationwide population‐based cohort study. Medicine (Baltimore). 2017;96:e5983. 28151890 10.1097/MD.0000000000005983 PMC5293453

[14] Wu Y , Sang Z‐Q , Zhang X‐C , Margraf J . The relationship between resilience and mental health in chinese college students: a longitudinal cross‐lagged analysis. Front Psychol. 2020;11:108. 32116918 10.3389/fpsyg.2020.00108 PMC7012791

[15] Bjelland I , Dahl AA , Haug TT , Neckelmann D . The validity of the Hospital Anxiety and Depression Scale. An updated literature review. J Psychosom Res. 2002;52:69–77. 11832252 10.1016/s0022-3999(01)00296-3

[16] Olsson I , Mykletun A , Dahl AA . The Hospital Anxiety and Depression Rating Scale: a cross‐sectional study of psychometrics and case finding abilities in general practice. BMC Psychiatry. 2005;5:46. 16351733 10.1186/1471-244X-5-46 PMC1343544

[17] Zigmond AS , Snaith RP . The hospital anxiety and depression scale. Acta Psychiatr Scand. 1983;67:361–70. 6880820 10.1111/j.1600-0447.1983.tb09716.x

[18] Christensen AV , Dixon JK , Juel K , Ekholm O , Rasmussen TB , Borregaard B , et al. Psychometric properties of the Danish Hospital Anxiety and Depression Scale in patients with cardiac disease: results from the DenHeart survey. Health Qual Life Outcomes. 2020;18:9. 31910859 10.1186/s12955-019-1264-0 PMC6947856

[19] Montazeri A , Vahdaninia M , Ebrahimi M , Jarvandi S . The Hospital Anxiety and Depression Scale (HADS): translation and validation study of the Iranian version. Health Qual Life Outcomes. 2003;1:14. 12816545 10.1186/1477-7525-1-14 PMC161819

[20] Abdollahi Z , Taghizadeh F , Zarghami M . The epidemiology of phosphine self‐poisoning in Sari, Iran, 2008–2010. Middle East J Sci Res. 2013;14:1180–3.

[21] Lee SM , Kang WS , Cho A‐R , Kim T , Park JK . Psychological impact of the 2015 MERS outbreak on hospital workers and quarantined hemodialysis patients. Compr Psychiatry. 2018;87:123–7. 30343247 10.1016/j.comppsych.2018.10.003 PMC7094631

[22] Shi H , Han X , Jiang N , Cao Y , Alwalid O , Gu J , et al. Radiological findings from 81 patients with COVID‐19 pneumonia in Wuhan, China: a descriptive study. Lancet Infect Dis. 2020. 10.1016/S1473-3099(20)30086-4 PMC7159053 32105637

[23] Gao J , Zheng P , Jia Y , Chen H , Mao Y , Chen S , et al. Mental health problems and social media exposure during COVID‐19 outbreak. PLoS One. 2020;15:e0231924. 32298385 10.1371/journal.pone.0231924 PMC7162477

[24] Jeong H , Yim HW , Song Y‐J , Ki M , Min J‐A , Cho J , et al. Mental health status of people isolated due to Middle East Respiratory Syndrome. Epidemiol Health. 2016;38. 10.4178/epih.e2016048 PMC5177805 28196409

[25] Pourali F , Afshari M , Alizadeh‐Navaei R , Javidnia J , Moosazadeh M , Hessami A . Relationship between blood group and risk of infection and death in COVID‐19: a live meta‐analysis. New Microbes and New Infections. 2020;37:100743. 10.1016/j.nmni.2020.100743 32837730 PMC7418722

[26] Rajkumar RP . COVID‐19 and mental health: A review of the existing literature. Asian J Psychiatr. 2020;52:102066. 32302935 10.1016/j.ajp.2020.102066 PMC7151415

[27] Huang Y , Zhao N . Generalized anxiety disorder, depressive symptoms and sleep quality during COVID‐19 outbreak in China: a web‐based cross‐sectional survey. Psychiatry Res. 2020;288:112954. 32325383 10.1016/j.psychres.2020.112954 PMC7152913

[28] Mo Y , Deng L , Zhang L , Lang Q , Liao C , Wang N , et al. Work stress among Chinese nurses to support Wuhan for fighting against the COVID‐19 epidemic. J Nurs Manag. 2020. 10.1111/jonm.13014 PMC7262235 32255222

[29] Zandifar A , Badrfam R , Yazdani S , Arzaghi SM , Rahimi F , Ghasemi S , Khamisabadi S , Mohammadian Khonsari N , Qorbani M . Prevalence and severity of depression, anxiety, stress and perceived stress in hospitalized patients with COVID‐19. Journal of Diabetes & Metabolic Disorders. 2020. 10.1007/s40200-020-00667-1 PMC7594988 33145259

[30] Sim K , Huak Chan Y , Chong PN , Chua HC , Wen Soon S . Psychosocial and coping responses within the community health care setting towards a national outbreak of an infectious disease. J Psychosom Res. 2010;68:195–202. 20105703 10.1016/j.jpsychores.2009.04.004 PMC7094450

[31] Seyed Hosseini E , Riahi Kashani N , Nikzad H , Azadbakht J , Hassani Bafrani H , Haddad Kashani H . The novel coronavirus Disease‐2019 (COVID‐19): Mechanism of action, detection and recent therapeutic strategies. Virology. 2020;551:1–9. 10.1016/j.virol.2020.08.011 33010669 PMC7513802

[32] Nukui H , Murakami M , Midorikawa S , Suenaga M , Rokkaku Y , Yabe H , et al. Mental health and related factors of hospital nurses: an investigation conducted 4 years after the Fukushima disaster. Asia Pacific J Public Health. 2017;29:161S–170S. 10.1177/1010539516682589 28330404

[33] Zhou C , Shi L , Gao L , Liu W , Chen Z , Tong X , et al. Determinate factors of mental health status in Chinese medical staff: A cross‐sectional study. Medicine (Baltimore). 2018;97:e0113. 29517690 10.1097/MD.0000000000010113 PMC5882452

[34] Nukui H , Midorikawa S , Murakami M , Maeda M , Ohtsuru A . Mental health of nurses after the Fukushima complex disaster: a narrative review. J Radiat Res. 2018;59:ii108–ii113. 29668971 10.1093/jrr/rry023 PMC5941163

[35] Zhu J , Sun L , Zhang L , Wang H , Fan A , Yang B , Xiao S , Li W . Prevalence and Influencing Factors of Anxiety and Depression Symptoms in the First‐Line Medical Staff Fighting Against the COVID‐19 in Gansu. Available at SSRN 3550054 2020. 10.3389/fpsyt.2020.00386 PMC7202136 32411034

[36] Zarghami M , Taghizadeh F , Moosazadeh M , Kheradmand M , Heydari K . Validity of self‐reporting depression in the Tabari cohort study population. Neuropsychopharmacology Reports. 2020;40(4):342–347. 10.1002/npr2.12138 32951353 PMC7722659

[37] Zandifar A , Badrfam R . Iranian mental health during the COVID‐19 epidemic. Asian J Psychiatr. 2020;51:101990. 32163908 10.1016/j.ajp.2020.101990 PMC7128485

[38] Nguyen HC , Nguyen MH , Do BN , Tran CQ , Nguyen TTP , Pham KM , et al. People with Suspected COVID‐19 Symptoms Were More Likely Depressed and Had Lower Health‐Related Quality of Life: The Potential Benefit of Health Literacy. Journal of Clinical Medicine. 2020;9(4):965. 10.3390/jcm9040965 PMC7231234 32244415

